# New Tags for Recombinant Protein Detection and O-Glycosylation Reporters

**DOI:** 10.1371/journal.pone.0096700

**Published:** 2014-05-06

**Authors:** Gianluca Petris, Marco Bestagno, Francesca Arnoldi, Oscar R. Burrone

**Affiliations:** 1 International Centre for Genetic Engineering and Biotechnology (ICGEB), Trieste, Italy; 2 Department of Medicine, Surgery and Health Sciences, University of Trieste, Trieste, Italy; Wadsworth Center, New York State Dept. Health, United States of America

## Abstract

Monoclonal antibodies (mAbs), because of their unique specificity, are irreplaceable tools for scientific research. Precise mapping of the antigenic determinants allows the development of epitope tagging approaches to be used with recombinant proteins for several purposes. Here we describe a new family of tags derived from the epitope recognized by a single highly specific mAb (anti-roTag mAb), which was obtained from a pool of mAbs reacting with the rotavirus nonstructural protein 5 (NSP5). The variable regions of the anti-roTag mAb were identified and their binding capacity verified upon expression as a single-chain/miniAb. The minimal epitope, termed roTag, was identified as a 10 amino acid sequence (SISSSIFKNE). The affinity of the anti-roTag/roTag interaction was found to be comparable to that of the anti-SV5/SV5 tag interaction. roTag was successfully used for detection of several recombinant cytosolic, secretory and membrane proteins. Two additional variants of roTag of 10 and 13 amino acids containing O-glycosylation susceptible sites (termed OG-tag and roTagO) were constructed and characterised. These tags were useful to detect proteins passing through the Golgi apparatus, the site of O-glycosylation.

## Introduction

In biological sciences development of new specific monoclonal antibodies (mAbs) is a pressing requirement for several aspects in the field: from basic research on protein function, to medical diagnosis, prophylaxis and therapy of several pathogenic conditions [1], [2], [3], [4], [5], [6]. Taking advantage of the hybridoma technology to produce monoclonal antibodies of desired specificity [7], [8], a number of mAb/epitopes pairs derived from different proteins have been characterized and used as tags to facilitate identification of recombinant proteins. Indeed, epitope tagging is a common methodology used to identify recombinant proteins when specific antibodies for the protein of interest are not readily available [9]. This technique consists in the expression of fusion proteins, obtained by inserting a nucleotide sequence encoding a peptide tag into the gene of interest. Usually a peptide tag is a short peptidic sequence (an epitope) recognized by an already existing antibody [10]. Tags can be used for protein detection in immunoenzymatic or immunochemical assays, as well as for protein isolation and purification by immunoprecipitation or affinity chromatography [11], [12]. Epitope tagging can help in the characterization of the tagged protein, by facilitating the determination of its abundance, cellular location, post-translational modifications, interactions with other proteins, etc. In addition, if the tag-specific antibody displays differential affinity depending on different post-translational modifications (e.g. phosphorylation or glycosylation) on the tag sequence itself, this can be exploited, for instance, to obtain information about activation status [13] or trafficking of the tagged protein through cellular compartments where those modifications take place [14]. Epitope tagging offers a number of advantages over alternative detection and purification methods, because it saves time and resources comparing with the traditional methods for producing specific antibodies (either monoclonal or polyclonal) to the protein of interest.

As tags are often short (6–15 amino acids in length), they are generally presumed to have no effect on the biological functions of the tagged proteins. However, if located in inappropriate positions, they might interfere with protein structure, function and interactions. In addition, not all mAb are suitable for every immunodetection method, as in the case of mAb specific for non-linear epitopes. For those reasons, it is useful to develop mAbs and epitope tags of different sequence characteristics (size, net charges, hydrophobicity and side groups) or that can be fused in different positions of the target protein to increase the chances of success in tagging applications.

Here we describe and characterize a new 10 amino acids long epitope tag (roTag) derived from the sequence of the rotavirus (RV) non-structural protein 5 (NSP5). NSP5 has an essential role during the RV replication cycle, as it is essentially required for the assembly of viroplasms, the sites of viral genome replication and initial assembly of progeny virus [15], [16]. In this context, since the precise role of NSP5 is still poorly understood [17], [18], we developed a series of novel mAbs reacting with different NSP5 domains. One highly specific anti-NSP5 mAb (1F2/anti-roTag) was identified and the recognized minimal linear epitope was mapped. The epitope, termed roTag, was shown to be highly specific when fused to reporter proteins. Further variants of roTag have been derived, including an O-glycosylation site, that proved useful to determine whether proteins in the secretory pathway have trafficked through the Golgi, according to their O-glycosylation status.

## Results and Discussion

### Characterization of anti-roTag mAb

**Figure 1:**
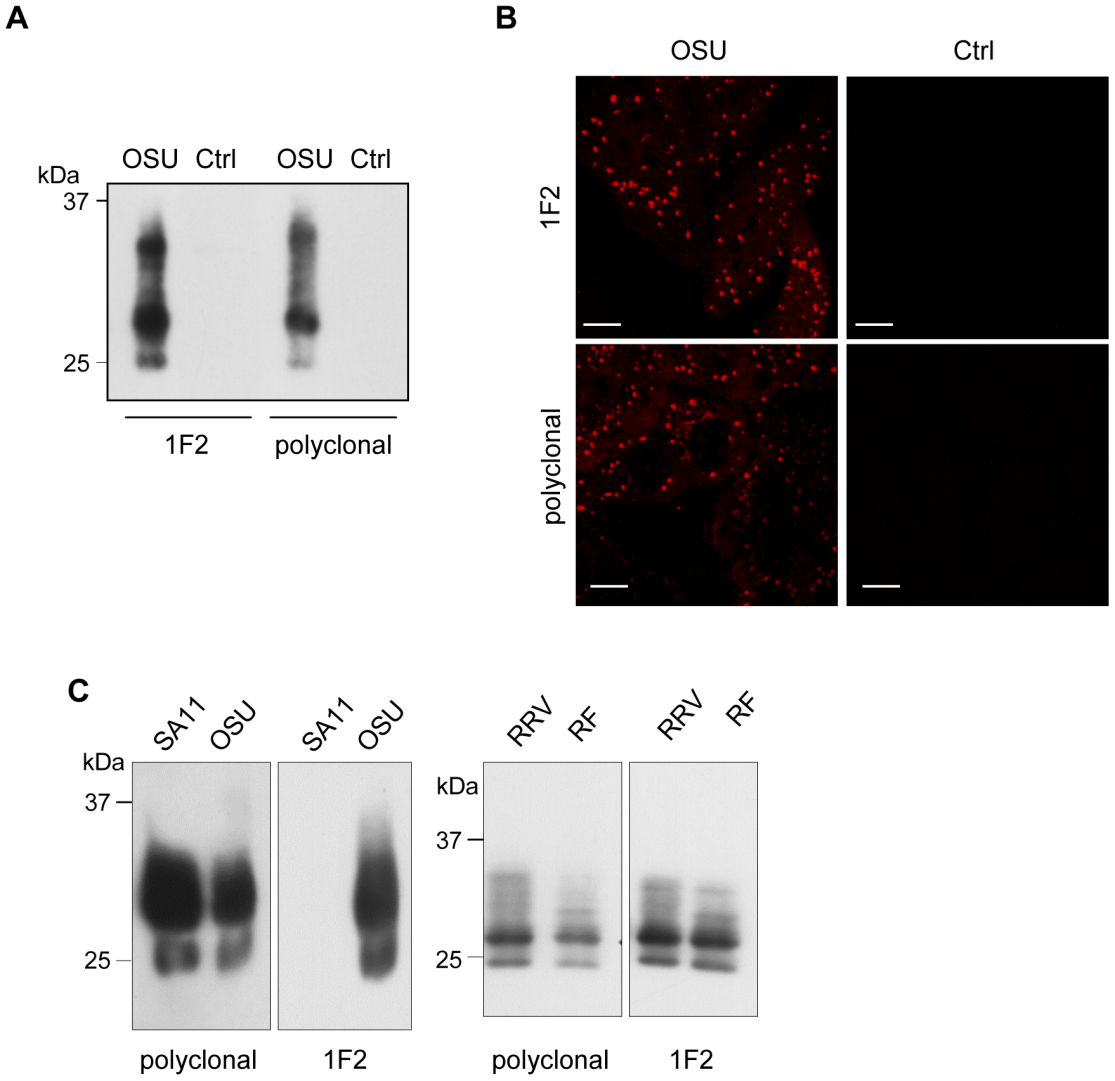
mAb 1F2 recognizes NSP5 (OSU strain) isoforms in cell extracts and in viroplasms. (A) WB of cellular extracts of RV-infected (OSU) or control (Ctrl) MA104 cells, as indicated. WB was developed with polyclonal anti-NSP5 or 1F2. (B) Confocal immunofluorescence of RV-infected or non-infected MA104 cells reacted with the primary antibodies used in (A); scale bar 10 mm. (C) WB of cellular extracts of MA104 cells infected with RV strains SA11, OSU, RRV and RF and developed as indicated.

A panel of anti-NSP5 mAbs were generated from BALB/c mice immunized with a Ni^++^-purified His-tagged NSP5 protein of the RV porcine OSU strain [19]. Screening of more than 400 clones by ELISA yielded 20 positive clones, of which 6 were further confirmed positive in RV-infected cells. mAb 1F2 (IgG1γ isotype) was selected because of its stronger reactivity, comparable to that of a polyclonal immune serum, in both IF staining of viroplasms in virus-infected cells (Figure 1B) and Western blot (WB) detection of essentially all the NSP5 phosphorylation isoforms (from 26 to 34 kDa) (Figure 1A). A full gel of cellular extracts of non-infected and RV-infected MA104 cells developed with 1F2 (high exposure) showed no cross-reactivity with cellular or viral proteins other than NSP5 isoforms (Figure S1A). Of note, mAb 1F2 showed strain specificity as it was unable to reveal NSP5 from simian SA11 strain as the control polyclonal anti-NSP5 serum, while NSP5 isoforms from two other RV strains, simian RRV and bovine RF, were efficiently detected (Figure 1C).

### Mapping the mAb 1F2 NSP5 epitope

**Figure 2:**
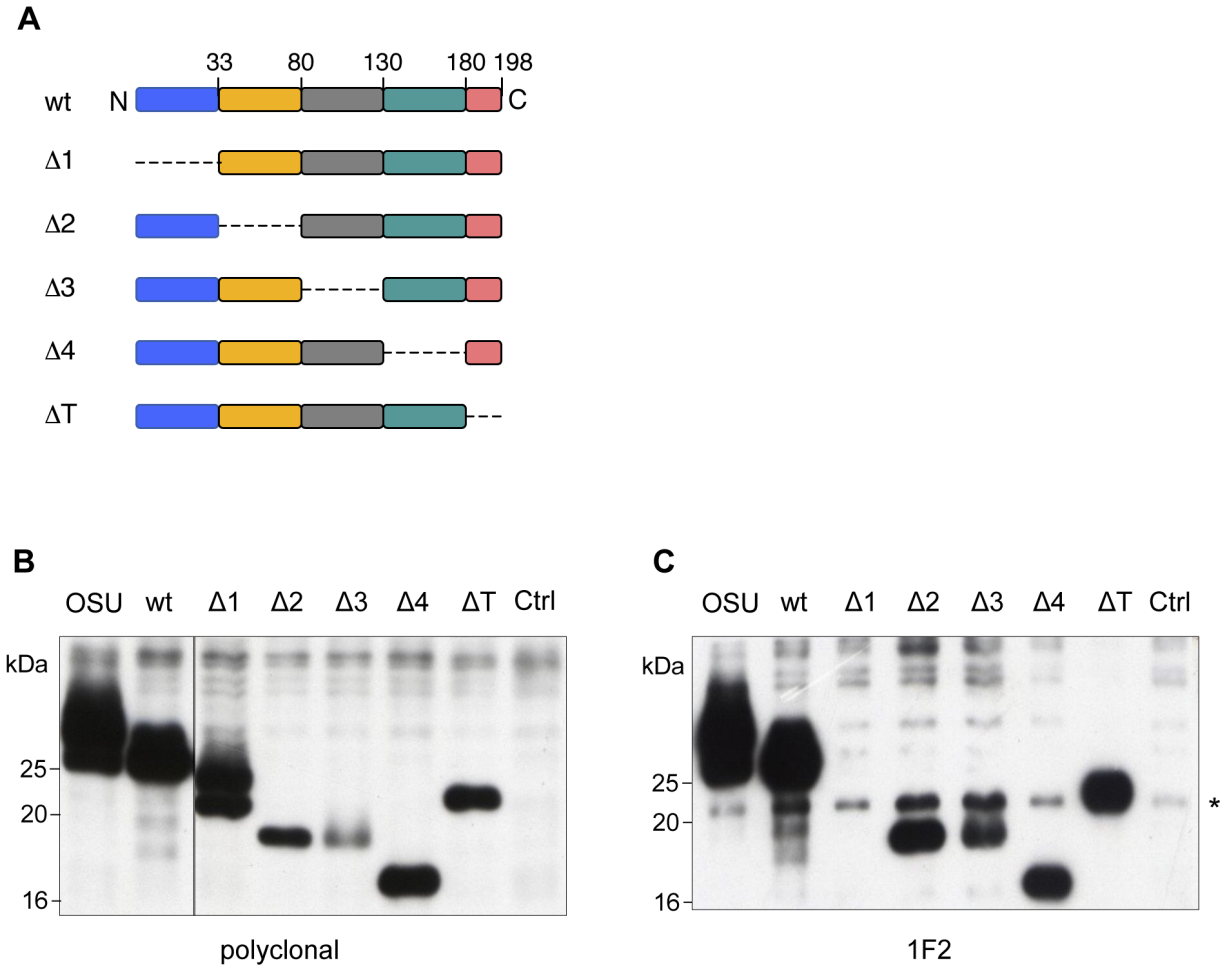
mAb 1F2 epitope maps in the N-terminal portion of NSP5. (A) Scheme of NSP5 deletion mutants. (B) and (C) WB of cellular extracts of HEK 293T cells infected with RV (OSU, lane 1) or transfected with constructs encoding full-length NSP5 (wt), NSP5 deletion mutants or empty vector (Ctrl). WB were developed with polyclonal anti-NSP5 serum (B) or 1F2 (C). In (C) lanes corresponding to mutants Δ2 and Δ3 were overloaded as both mutants were expressed at lower levels. Asterisk indicates a 21kDa non-specific band.

To establish the epitope on NSP5 we took advantage of a series of NSP5 deletion mutants [20], [21] shown in Figure 2A. Plasmids encoding mutants NSP5-Δ1, NSP5-Δ2, NSP5-Δ3, NSP5-Δ4 and NSP5-ΔT were transfected in HEK 293T cells and tested with mAb 1F2 or with a polyclonal anti-NSP5 immune serum. As shown in Figure 2B, all the deletion mutants were expressed in HEK 293T cells with reduced hyperphosphorylation, as expected for NSP5 expressed in mammalian cells in the absence of other viral proteins (Figure 2B lanes 1 and 2) [20], [21]. mAb 1F2 recognised all mutants with the exception of NSP5-Δ1, indicating the localization of the epitope within the first N-terminal 33 aa (Figure 2C).

**Figure 3:**
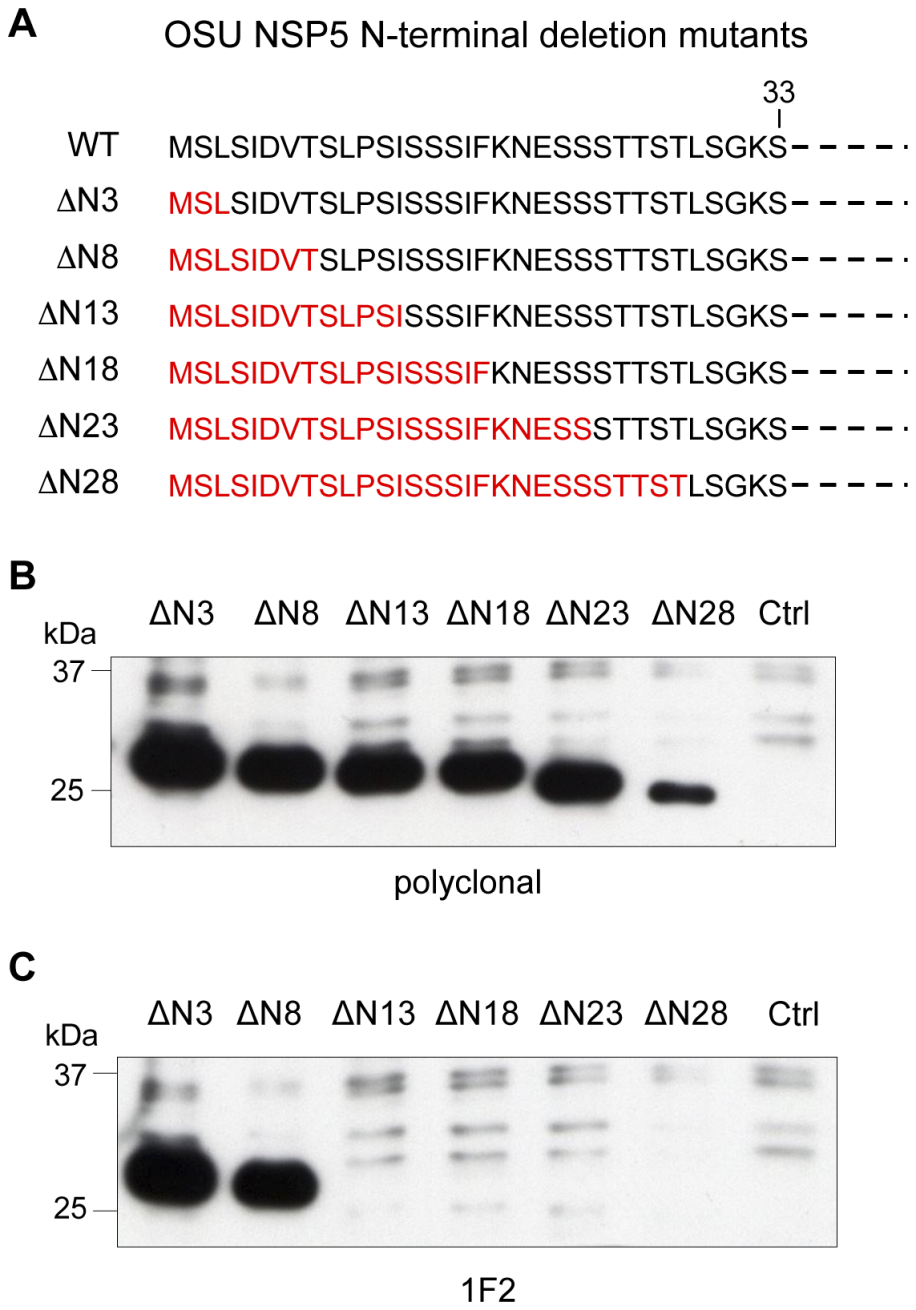
mAb 1F2 detection of NSP5 N-terminal deletion mutants. (A) NSP5 N-terminal sequence; deleted amino acids are indicated in red. (B) and (C) WB of cellular extracts of HEK 293T cells transfected with NSP5 N-terminal deletion mutants or empty vector (Ctrl), developed with polyclonal anti-NSP5 serum (B) or 1F2 (C).

To further define the epitope, a series of progressive N-terminal deletion mutants, NSP5-ΔN3, NSP5-ΔN8, NSP5-ΔN13, NSP5-ΔN18, NSP5-ΔN23 and NSP5-ΔN28, were assayed with a polyclonal anti-NSP5 serum or with 1F2 by WB. As shown in Figure 3, while all deletion mutants were expressed and detected by the anti-NSP5 serum (Figure 3B), 1F2 revealed only NSP5-ΔN3 and NSP5-ΔN8 (Figure 3C), thus indicating that the determinant initiates between Ser9 and Ile13.

**Figure 4:**
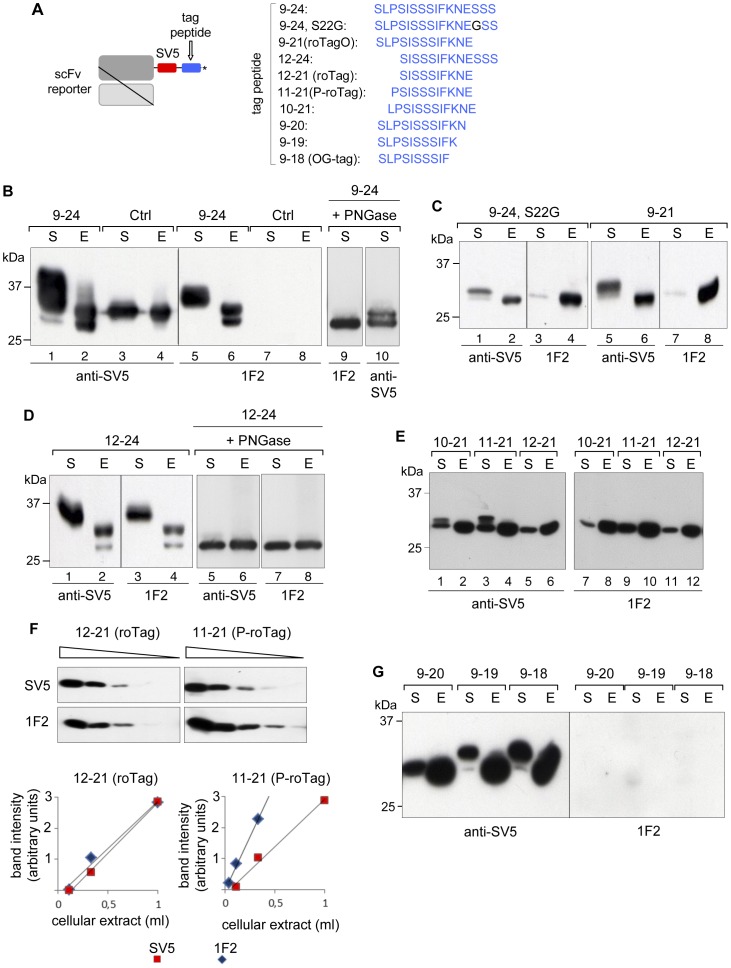
Mapping of mAb 1F2 epitope and related tags. (A) Scheme of the reporter protein used (scFv) tagged with the peptides shown in the right panel. The amino acid sequence present in NSP5 is highlighted in blue. (B) – (E) WB of cellular extracts (indicated as E) and supernatants (indicated as S) of HEK 293T cells transfected with the reporter protein tagged with: (B) an irrelevant amino acid sequence (Ctrl) or peptide 9–24, (C) peptides 9–24, S22G or 9–21, (D) peptide 12–24, (E) peptides 10–21, 11–21 or 12–21, (F) peptides 11–21 or 12–21 (here also indicated as P-roTag and roTag, respectively), (G) peptides 9–20, 9–19 and 9–18. In all panels blots were developed, as indicated, with anti-SV5 or 1F2. (F) Equal dilutions of cell extracts used in (E) analyzed by WB and densitometric analysis. The plot shows values for the non-saturating linear range part.

We then used a secretory scFv as a reporter protein [22], tagged with various peptide sequences comprised between amino acids 9 and 24 (Fig 4A). The 11 amino acids long SV5 tag was also included. As shown in Figure 4B, two isoforms of the reporter protein tagged with the 9–24 peptide were detected in cell extracts by both anti-SV5 and 1F2 (lanes 2, 6), while, as expected, the reporter with a control irrelevant tag (Ctrl) was not revealed by 1F2 (lanes 7, 8). In addition, in the intracellular material a number of other isoforms with slower mobility were also detected by anti-SV5, but not by 1F2, suggesting post-translational modifications on the 9–24 peptide that compromised recognition by 1F2. This was even more clear in the secreted fraction, 1F2 recognised a single 35 kDa band with slower mobility than the intracellular bands (lane 5, 6), while a second slower mobility band was apparent with anti-SV5 (lane 1). Since a putative N-glycosylation site is present in position N20 (NES), it is possible that the two bands in the cell extracts represented glycosylated and non-glycosylated material, while the slower mobility band in the supernatant represented terminally N-glycosylated secreted molecules. This was confirmed by PNGase digestion (lanes 9 and 10) that converted the material in the supernatant to a single lower molecular mass band detected by anti-1F2 (lane 9), while an extra band was detected by anti-SV5 (lane 10), suggesting the presence of an additional post-translational modification. Further confirmation of N20 glycosylation was obtained mutating S22 into Gly in tag 9–24 (9–24,S22G), which caused total loss of the upper band on the cell extracts detected by both mAbs (Figure 4C, lanes 2, 4). Thus recognition by 1F2 was not affected by glycosylation of N20. In supernatants, however, anti-SV5 still detected a slower mobility band that was not detected by 1F2, confirming the additional post-translational modification, which disturbs recognition by 1F2 (Figure 4C lanes 1, 3). A faint band with the same mobility of the intracellular protein was detected by both mAbs, likely representing non-modified material. Essentially, the same distribution of bands was observed with the 9–21 peptide tag (Figure 4C, lanes 5–8). Thus, the reporter was secreted mostly as a post-translationally modified protein and only a small proportion without such modification (still detected by 1F2). This second post-translational modification was likely an O-glycosylation, taking place in the secretory pathway while in transit through the Golgi complex, as it was mainly present in the secreted material. Sequences rich in serines with a proline in position -1/-3 favor O-glycosylation of those residues [23], [24], and such a conformation is present in peptide 9–21 (SL**PS**I**S**SSIFKNE) (and also in 9–24) suggesting involvement of S12 and S14. In fact, a tag initiating in S12 (tag 12–24) resulted in a pattern of bands equally recognized by anti-SV5 and 1F2, both in the intracellular and in the secreted material (Figure 4D, lanes 1–4), indicating that in the absence of P11, O-glycosylation was not taking place, while N-glycosylation was still present. Indeed, following PNGase treatment a single band was detected with 1F2 and anti-SV5, both in cell extracts and in supernatants (Figure 4D, lanes 5–8). When peptides 10–21, 11–21 and 12–21 were assayed, both mAbs detected intracellularly a single band of the same mobility in all three cases (Figure 4E, lanes 2, 4, 6, and 8, 10, 12), while in the secreted material an identical pattern was observed with both mAbs only with peptide 12–21 (Figure 4E, lanes 5, 11). The slower mobility bands in the supernatants of tags 10–21 and 11–21, detected by anti-SV5 but not by 1F2 (lanes 1, 3 and 7, 9), represented post-translationally modified molecules. These results further confirm that P11 is crucial to induce the modification observed after secretion, consistent with O-glycosylation in S12 and/or S14 during transit through the Golgi. The full gel of Figure 4E, showing no cross reactivity with cellular proteins is shown in Figure S1B.

Recognition of tag 11–21 was apparently stronger than tag 12–21 indicating that P11 plays also a role in favoring 1F2 binding. To better analyze this point we serially diluted cell extracts containing the reporter protein tagged with 11–21 and 12–21 (Figure 4F) and compared detection with 1F2 and anti-SV5. While similar recognition was observed for the 12–21-tagged reporter with both mAbs, an apparent 2.5 fold higher affinity was observed for 1F2 on the 11–21-tagged protein. This observation also indicates that, while P11 is *per se* not essential in the 1F2 epitope, its presence improves detection, probably due to an increased rigidity introduced to the determinant.

Since I13 was an essential part of the epitope (mutant ΔN13 was not recognised) and because of the relevant impact of P11 on O-glycosylation, we defined S12 as the N-terminal border of the epitope and termed roTag the peptide 12–21 and P-roTag the one starting in P11 (11–21). The C-terminal border of the anti-roTag/1F2 epitope was confirmed to be E21, as peptides 9–18, 9–19 and 9–20 were not detected by anti-roTag (Figure 4G). All these tags that include P11 showed a decreased migration pattern of different extent in supernatants (detected by anti-SV5), compatible with O-glycosylation. This was particularly relevant for peptide 9–18, where almost all the secreted material showed a clear discrete change in electrophoretic mobility. This tag, therefore, not recognized by anti-roTag, but very efficiently O-glycosylated, was termed OG-tag. In contrast, the strong O-glycosylation sensitive tag 9–21 that contained the core roTag epitope was termed roTagO. Tags including P11 are functional to detect molecules that do not traffic through the Golgi, such as cytosolic, mitochondrial, nuclear or ER resident proteins. Table 1 summarizes the properties of all this four characterized tags.

**Table 1:** Sequences and properties of roTag epitopes family.

Tag Name	Sequence	Detection by 1F2	O-glycosylation
roTag	SISSSIFKNE	+	-
P-roTag	PSISSSIFKNE	++ if not O-glycosylated	+
roTagO	SLPSISSSIFKNE	++ if not O-glycosylated	++
OG-Tag	SLPSISSSIF	-	++

**Figure 5:**
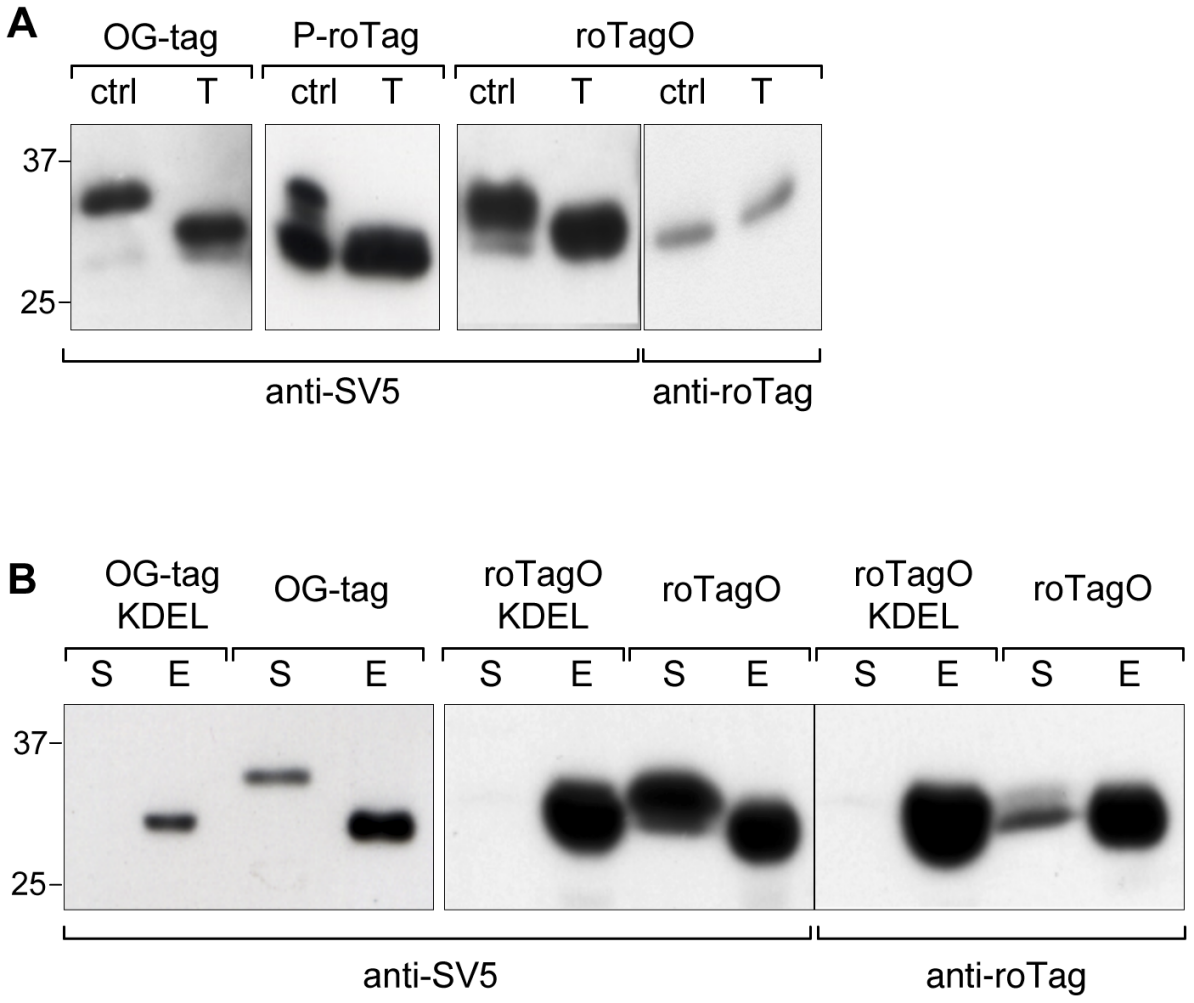
O-glycosylated tags. (A) WB of supernatants of 293T cells transfected with the reporter protein tagged with OG-tag, P-roTag or roTagO (previously indicated as 9–18, 11–21 and 9–21, respectively) treated (T) or not (Ctrl) with a glycosidase mix containing Neuraminidase, β1-3 Galactosidase and β-NAc-hexosaminidase. (B) WB of cellular extracts (E) and supernatants (S) of HEK 293T cells transfected with the reporter protein tagged with OG-tag and roTagO with or without the ER retention signal KDEL. In all panels blots were developed, as indicated, with anti-SV5 or anti-roTag/1F2.

O-glycosylation was confirmed for roTagO, P-roTag and OG-tag by treatment of supernatants containing the reporter protein (with either of the three tags) with a glycosidase cocktail. As shown in Figure 5A, when incubated with Neuraminidase, β1-3 Galactosidase and β-N-Ac-hexosaminidase enzymes (a cocktail that removes most of the sugars present in several O-glycosylation types [24]) the isoforms of higher apparent molecular mass were efficiently converted into faster migrating isoforms. Nevertheless, this cocktail was not sufficient to remove all sugars present, as detection by anti-roTag was not rescued after this treatment, confirming the high sensitivity of anti-roTag (1F2) to the O-glycosylated epitope (Figure 5A). Further demonstration that O-glycosylation was modifying roTagO and OG-tag was obtained introducing the sequence KDEL at the C-terminus of the reporter. KDEL is sufficient to prevent secretion of soluble proteins, because binding to KDEL receptors in the ER lumen causes retention in the ER compartment, thus blocking traffic through the Golgi [25]. As shown in Figure 5B, KDEL was *per se* sufficient to cause retention in the ER and to completely prevent O-glycosylation, according to three different criteria: unchanged mobility, lack of secretion and detection by mAb anti-roTag.

**Figure 6:**
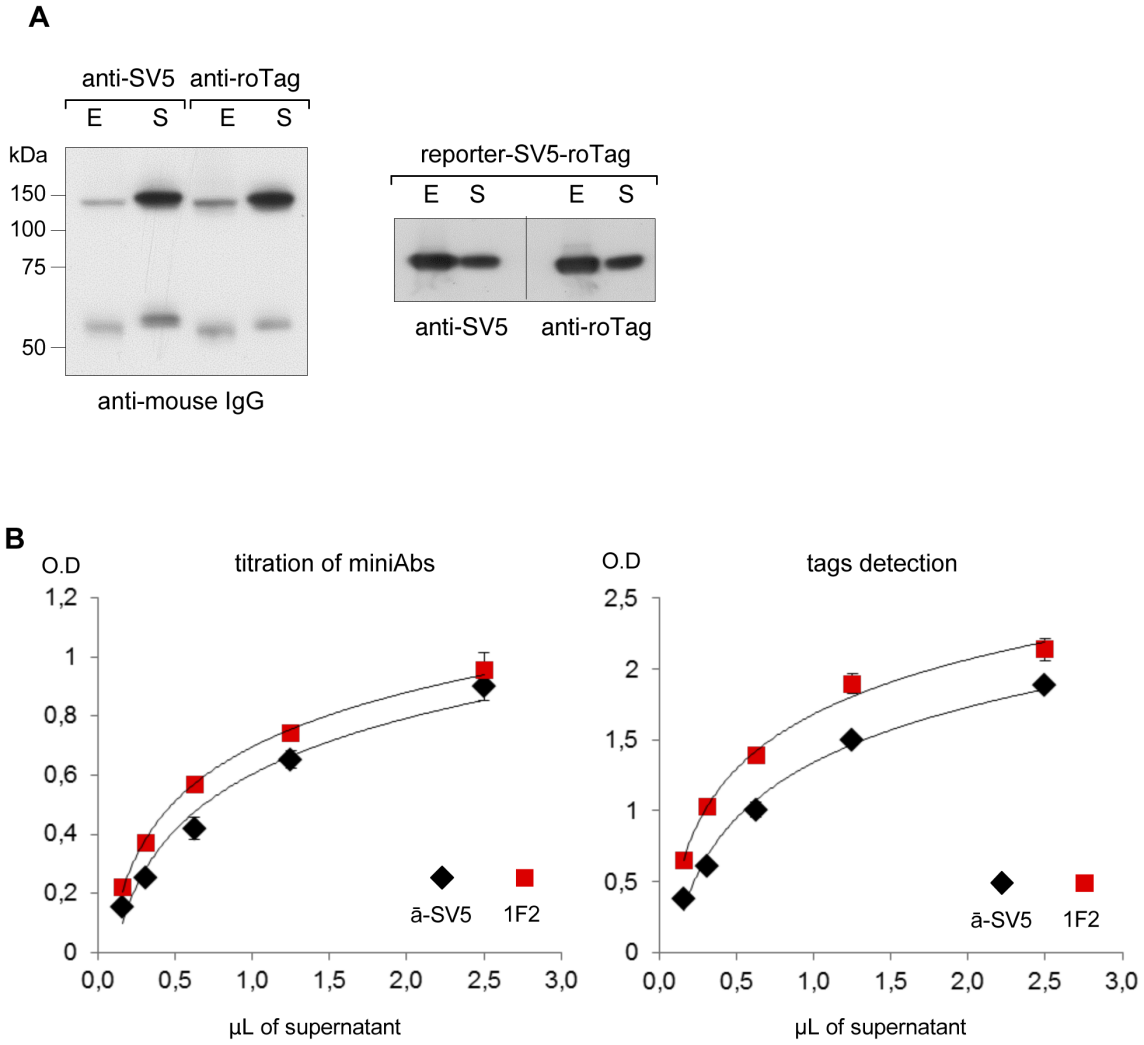
Comparable reactivity of anti-SV5 and anti-roTag. (A) Left panel, WB of cell extracts (E) and supernatants (S) of HEK 293T cells transfected with miniAb constructs encoding anti-SV5 and anti-roTag, developed with HRP-conjugated anti-mouse IgG. Right panel, WB of cell extracts and supernatants containing the double-tagged (SV5-roTag) reporter, developed with anti-SV5 and anti-roTag, as indicated. (B) Plot of ELISA results: left panel, relative quantification of anti-SV5 and anti-roTag concentrations; right panel, reactivity of anti-SV5 and anti-roTag on immobilised double-tagged (SV5 and roTag) reporter. ELISA experiments were performed in triplicate; error bars indicate standard deviation.

The relative reactivity of anti-roTag was then directly compared to the widely used anti-SV5 using recombinant versions of the two mAbs (anti-SV5 and anti-roTag) containing the scFv (V_L_-linker-V_H_) fused to the hinge-CH2-CH3 domains of the γH chain of murine IgG2b. The resulting recombinant antibodies (miniAb, [26]) showed comparable expression levels in transfected 293T cells (Figure 6A). Whilst in WB both mAbs were able to efficiently detect the double-tagged reporter, a more precise comparison of the reactivity was obtained by ELISA. As shown in Figure 6B, anti-roTag showed, at all concentrations, a comparable (even slightly higher) reactivity to anti-SV5.

## Conclusions

Here we described the characterization of four related new tags with defined properties. mAb anti-roTag/1F2 was elicited against RV NSP5 and resulted in a highly specific reagent that detects the complex pattern of NSP5 phosphorylated isoforms in WB and the viroplasms in virus-infected cells in IF experiments. mAb anti-roTag can be applied in ELISA, WB, IF, immunoprecipitation and cytofluorimetry ([27], [28], [29] and unpublished observations). In addition, it discriminates between different virus strains, as it recognizes NSP5 of strains OSU, RRV and RF, but not that of strain SA11, which differs in three residues in the roTag epitope (S14P, S16T, F18Y). Interestingly, since RRV NSP5 sequence shares with SA11 the mutation F18Y, and yet it is recognized by 1F2, Serines 14 and 16 can be critical for of 1F2 reactivity.

Characterisation of the anti-roTag epitope allowed us to define a series of four different tags: a) the 10 amino acids long roTag (peptide 12–21) that can be efficiently used with different proteins, in N-terminal, middle and C-terminal positions and in different cellular compartments (cytosol, ER lumen, secreted) ([27], [28], [29] and unpublished results); b) P-roTag (11 aa), a variant of roTag recognized with higher relative affinity, useful for proteins that do not traffic through the Golgi (like nuclear, cytosolic and ER resident proteins), since the extra proline at the N terminus activates the post-translational addition of O-linked sugars if the tagged protein reaches the Golgi complex, resulting in complete loss of recognition by anti-roTag; c) roTagO (13 aa), an N-terminal extended version of roTag (peptide 9–21), which shows very efficient Golgi-dependent O-glycosylation; this tag is a particularly useful tool to study traffic through the Golgi, since the precursor is well detected by anti-roTag, while the fraction reaching the Golgi becomes O-glycosylated and therefore undetectable; d) the 10 amino acids long OG-tag (peptide 9–18) not recognized by anti-roTag mAb, a convenient tag for Golgi-dependent O-glycosylation, visualized as a strong reduced mobility in PAGE. To date, a number of epitope tags have been reported, which are often incorporated into plasmid vectors for expression in mammalian, insect, plant, yeast or bacterial cells [30], [31], [32], [33], [34], [35], [36], [37], [38], [39], [40], [41], [42], [43], [44], [45], [46], [47]. None of them, however, have the unique characteristics of the roTag family here described.

## Materials and Methods

### Ethics statement

Animal care and treatments were conducted in conformity with institutional guidelines in compliance with national and international laws and policies (EEC Council Directive 86/609, OJL 358, December 12, 1987 and D.lgs 116/92), approved by the ICGEB Trieste Ethics Committee for Animal Experimentation. Animals were anesthetized (isoflurane) before venipuncture and sacrifice (asphyxiation in carbon dioxide). All efforts were made to minimize suffering.

### Immunization and mAb production

Balb/c mice have been immunized with Ni^++^-purified His-tagged NSP5 protein [19], by five injections with 50 µg of purified protein in incomplete Freund adjuvant emulsion. Lymphocytes from spleen of immunized animal were fused with Sp2/0 myeloma cells using polyethylene glycol and hybridoma clones isolated by the HAT selection method, according to standard procedures [48]. Clones expressing anti-NSP5 antibodies were selected by ELISA of supernatants on purified NSP5 protein and the specificity of the secreted antibodies was confirmed by immunofluorescence on RV-infected cells and by Western blotting on lysates from RV-infected cells.

### Constructs

NSP5 deletion mutants were previously described [20], [21]. NSP5 N terminus deletion mutants were obtained by PCR and cloned into pcDNA3 vector via KpnI and BamHI digestion. RNA from anti-roTag hybridoma was purified using RNeasy mini kit (Qiagen), following manufacturer instructions.

After cDNA synthesis VL and VH were amplified and cloned in pUC18 using primers VKB-2 5′: 5′-GATATTGTGATGACCCAGTCTCCA-3′, CK-2 3′: 5′-TGGATACAGTTGGTGCAGC-3′, VHS-2 5′: 5′-TGTGCACTCYSAGGTSMARCT-3′, CHγ 3′: 5′-GGCCAGTGGATAGAC-3′. From pUC18 V_L_ and V_H_ regions were amplified with primers 1F2-VL-for: 5′TATGGTGCACTCTGATGTTGTGATGACCCAGACTCCA-3′, 1F2-VL-rev: 5′-TATAACTAGTGCTGCCTTTCAGCTCCAGCTTGGT-3′, VH-for 5′: 5′-TCTCTCGAGCAAAGGTCAGGTCCAACTGCAGCAGTC-3′, 1F2-VH rev: 5′-AGTTCCGGAGGAGACGGTGACTGA-3′ and cloned ApaLI/BspEI into pUT vector downstream of a leader peptide [49] and then together with the leader peptide by HindIII/BspEI into pcDNA3 upstream of domains Hinge C_H_2 e C_H_3 of IgG2b, which were previously amplified from murine splenocytes and inserted in pcDNA3 by BspEI/XbaI (primers MHG2B-1 5′: 5′-CCCTCCGGACCCATTTCAACAATCA-3′ and MHG2B-2 3′: 5′-TCTTCTAGAGCTCATTTACCCGGAGA-3′).

For mapping the anti-roTag epitope the following synthetic oligonucleotides encoding the different epitope tags were cloned as BamHI/EcoRI, downstream of SV5 in a plasmid containing scFv-SV5-BAP previously described [22], 9-24-1 5′: 5′-GATCCCTTCCCTCAATTTCTTCTAGTATCTTTAAAAATGAATCG

TCTTCTTAAG-3′, 9-24-2 3′: 5′-AATTCTTAAGAAGACGATTCATTTTTAAAGATACTAGAAGAAA

TTGAGGGAAGG-3′, 9-24G-1 5′: 5′-GATCCCTTCCCTCAATTTCTTCTAGTATCTTTAAAAATGAAGG

TTCTTCTTAAG-3′, 9-24G-2 3′: 5′-AATTCTTAAGAAGAACCTTCATTTTTAAAGATACTAGAAGAAATTGAGGGAAGG-3′, 9-21-1 5′: 5′-GATCCCTTCCCTCAATTTCTTCTAGTATCTTTAAAAATGAAGGTTAAG-3′, 9-21-2 3′: 5′-AATTCTTAACCTTCATTTTTAAAGATACTAGAAGAAATTGAGGGAAGG-3′, 12-24-1 5′: 5′-GATCCATTTCTTCTAGTATCTTTAAAAATGAATCGTCTTCTTAAG-3′, 12-24-2 3′: 5′-AATTCTTAAGAAGACGATTCATTTTTAAAGATACTAGAAGAAATG-3′, 9-16-1 5′: 5′-GATCCCTTCCCTCAATTTCTTCTAGTTAAG-3′, 9-16-2 3′: 5′-AATTCTTAACTAGAAGAAATTGAGGGAAGG-3′, 10-21-1 5′: 5′-GATCCGGTGGCCTTCCCTCAATTTCTTCTAGTATCTTTAAAAATGAAGGTTAAG-3′, 10-21-2 3′: 5′-AATTCTTAACCTTCATTTTTAAAGATACTAGAAGAAATTGAGGGAAGGCCACCG-3′, 11-21-1 5′: 5′-GATCCGGTGGCCCCTCAATTTCTTCTAGTATCTTTAAAAATGAAGGTTAAG-3′, 11-21-2 3′: 5′-AATTCTTAACCTTCATTTTTAAAGATACTAGAAGAAATTGAGGGGCCACCG-3′, 12-21-1 5′: 5′-GATCCATTTCTTCTAGTATCTTTAAAAATGAAGGTTAAG-3′ 12-21-2 3′: 5′-AATTCTTAACCTTCATTTTTAAAGATACTAGAAGAAATG-3′, 9-20-1 5′: 5′-GATCCCTTCCCTCAATTTCTTCTAGTATCTTTAAAAATTAAG-3′, 9-20-2 3′: 5′-AATTCTTAATTTTTAAAGATACTAGAAGAAATTGAGGGAAGG-3′, 9-19-1 5′: 5′-GATCCCTTCCCTCAATTTCTTCTAGTATCTTTAAATAAG-3′, 9-19-2 3′: 5′-AATTCTTATTTAAAGATACTAGAAGAAATTGAGGGAAGG-3′, 9-18-1 5′: 5′-GATCCCTTCCCTCAATTTCTTCTAGTATCTTTTAAG-3′, 9-18-2 3′: 5′-AATTCTTAAAAGATACTAGAAGAAATTGAGGGAAGG-3′, roTagG KDEL-1: GATCCCTTCCCTCAATTTCTTCTAGTATCTTTAAAAATGAAGGTAAGGATGAGCTTTAAG, roTagG KDEL-2: AATTCTTAAAGCTCATCCTTACCTTCATTTTTAAAGATACTAGAAGAAATTGAGGGAAGG, OG-Tag KDEL-1: GATCCCTTCCCTCAATTTCTTCTAGTATCTTTGGAAAGGATGAGCTGTAAG, OG-Tag KDEL-2: AATTCTTACAGCTCATCCTTTCCAAAGATACTAGAAGAAATTGAGGGAAGG.

### Cell culture, transfection and infection

Sp2/0 myeloma cells (ATCC CRL-1581) were cultured in RPMI 1640 supplemented with 10% fetal calf serum (FCS); hybridoma clones were grown in the same medium supplemented with 2% Hybridoma Media Supplement (Sigma-Aldrich) and 1 mM sodium pyruvate. MA104 and HEK 293T cells were grown in Dulbecco's modified Eagle's medium (DMEM), supplemented with 10% fetal calf serum (FCS). Cells were co-transfected in 6-well plates (about 5×10^5^ cells/well) by standard calcium phosphate technique [50], 18 hours after transfection, medium was discarded and replaced by 2 ml of serum free medium for 24 hours. Simian SA11 (G3, P6 [1]), porcine OSU (G5, P9 [7]) bovine RF (G6, P6 [1]) and simian RRV (G6, kind gift from Dr. Eichwald, University of Zurich) strains were propagated in MA104 cells, as described previously [51], [52].

### Cells extract preparation, WB, IF, and ELISA

293T transfected cells were lysed with 100 µl of TNN lysis buffer (100 mM Tris-HCl pH 8.0, 250 mM NaCl, 0.5% NP40) supplemented with protease inhibitors cocktail (Sigma-Aldrich). In the experiments shown in Figures 3, 4, 5, 6, 10 µl of cell extract or 20 µl of the corresponding supernatants were denatured in SDS-gel-loading buffer (25 mM Tris HCl pH 6.8, 1% SDS, 10% glycerol, 175 mM β-mercaptoethanol), boiled for 10', separated on 10% SDS-PAGE and transferred to PVDF membrane for immunodetection with anti-SV5 (Invitrogen) or anti-roTag antibodies followed by incubation with HRP-labeled anti-mouse Igγ+μ (H+L) (KPL) and ECL reaction. Quantification of bands was performed with Image-J software. Where indicated samples were treated 2 hours at 37°C with a mix of Neuraminidase, β1-3 Galactosidase and β -N-Ac-hexosaminidase (New England Biolabs) in buffer 50 mM sodium citrate pH 4.5 according to manufacturer indications.

Immunofluorescence experiments were performed after cells were fixed in 3.7% paraformaldehyde in PBS for 10 min at room temperature. Coverslips were washed in PBS and blocked with 1% bovine serum albumin (BSA) in PBS for 30 min and incubated with mouse anti-NSP5 serum (1:500) in PBS–1% BSA, supernatants of anti-roTag hybridoma for 1 h at room temperature. After three washes in PBS, the slides were stained for 45 min with rhodamine isothiocyanate-conjugated secondary antibody (Sigma), washed, and mounted with ProLong mounting medium (Molecular Probes). Samples were analyzed by confocal microscopy (Zeiss LSM510).

ELISA to determine relative antibody concentrations was performed by capturing supernatants containing Abs on polystyrene microplates (Nunc Maxisorp C96) coated with 0.1 µg/ml of goat anti-mouse gamma in buffer NaHCO_3_-Na_2_CO_3_ 50 mM, pH 9.5 (100 µl/well). After reaction with HRP-labeled goat anti-mouse gamma Fc (Jackson Immunoresearch) plates were developed with tetramethylbenzidine (TMB) reagent (Sigma) and blocked with H_2_SO_4_. O.D. at 450 nm was read on a BioRad microplate reader 550. ELISA comparison of relative affinity of recombinant anti-SV5 and anti-roTag was performed by capturing the double-tagged (SV5-roTag) reporter [29] on plates coated with the anti-idiotypic P3 mAb [53], [54]. Serial dilution of supernatants containing recombinant anti-SV5 or anti-roTag antibodies were applied, followed by HRP-labeled anti-mouse gamma Fc and developed as before. ELISA experiments were performed in triplicates.

## Supporting Information

Figure S1 (A) Full gel, of non-infected and RV-infected MA104 cell extracts reacted with mAb 1F2. (B) Full gel of lanes shown in Figure 4E, reacted with both anti-Sv5 and 1F2.
(TIF)
