# Supplementary figures and images for: New Tags for Recombinant Protein Detection and O-Glycosylation Reporters
Source: PLoS One. 2014 May 6;9(5):e96700. doi: 10.1371/journal.pone.0096700 (PMC4011882; doi:10.1371/journal.pone.0096700)

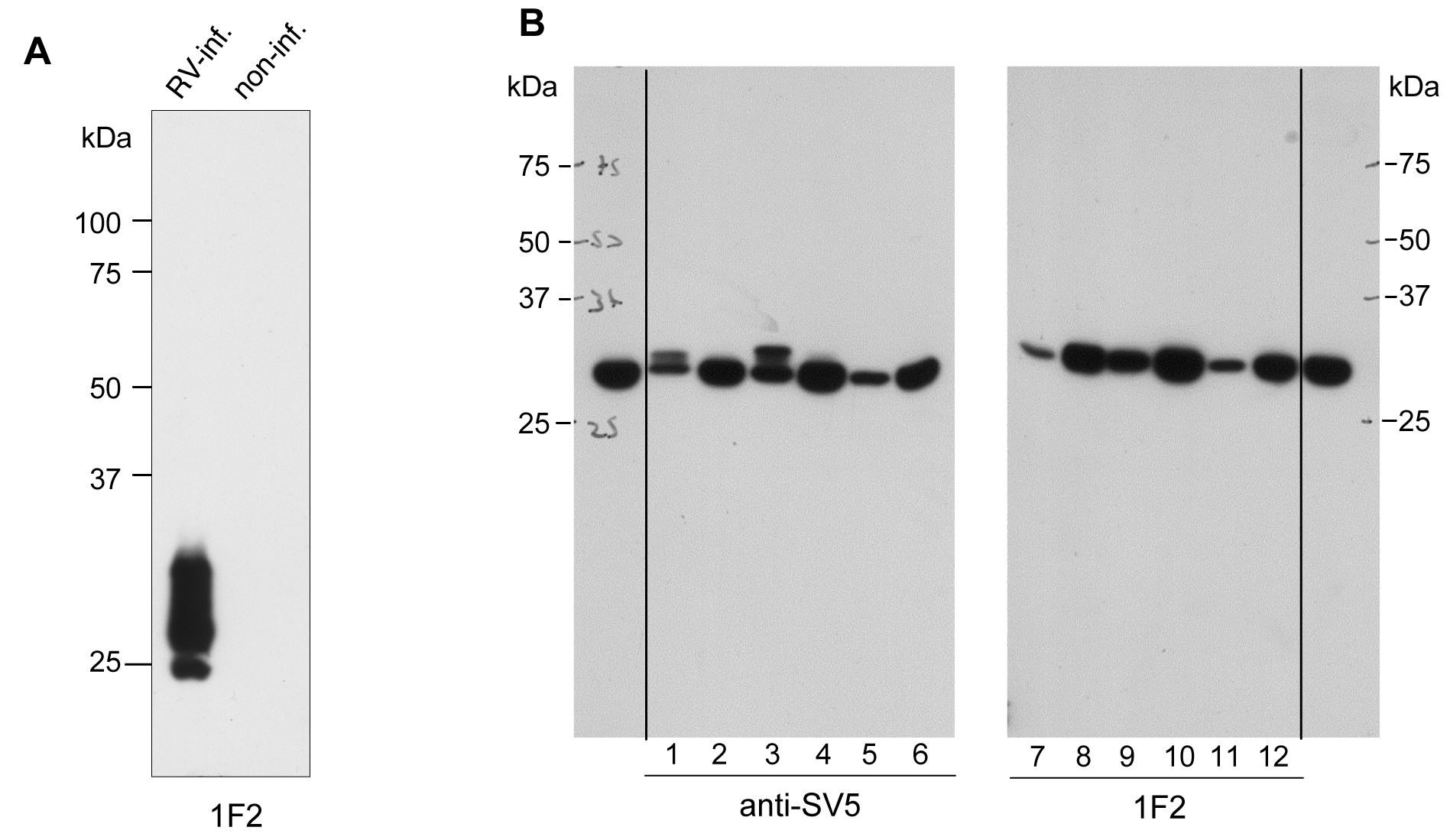

Supplement: Figure S1 — (A) Full gel, of non-infected and RV-infected MA104 cell extracts reacted with mAb 1F2. (B) Full gel of lanes shown in Figure 4E, reacted with both anti-Sv5 and 1F2. (TIF) [file pone.0096700.s001.tif]
